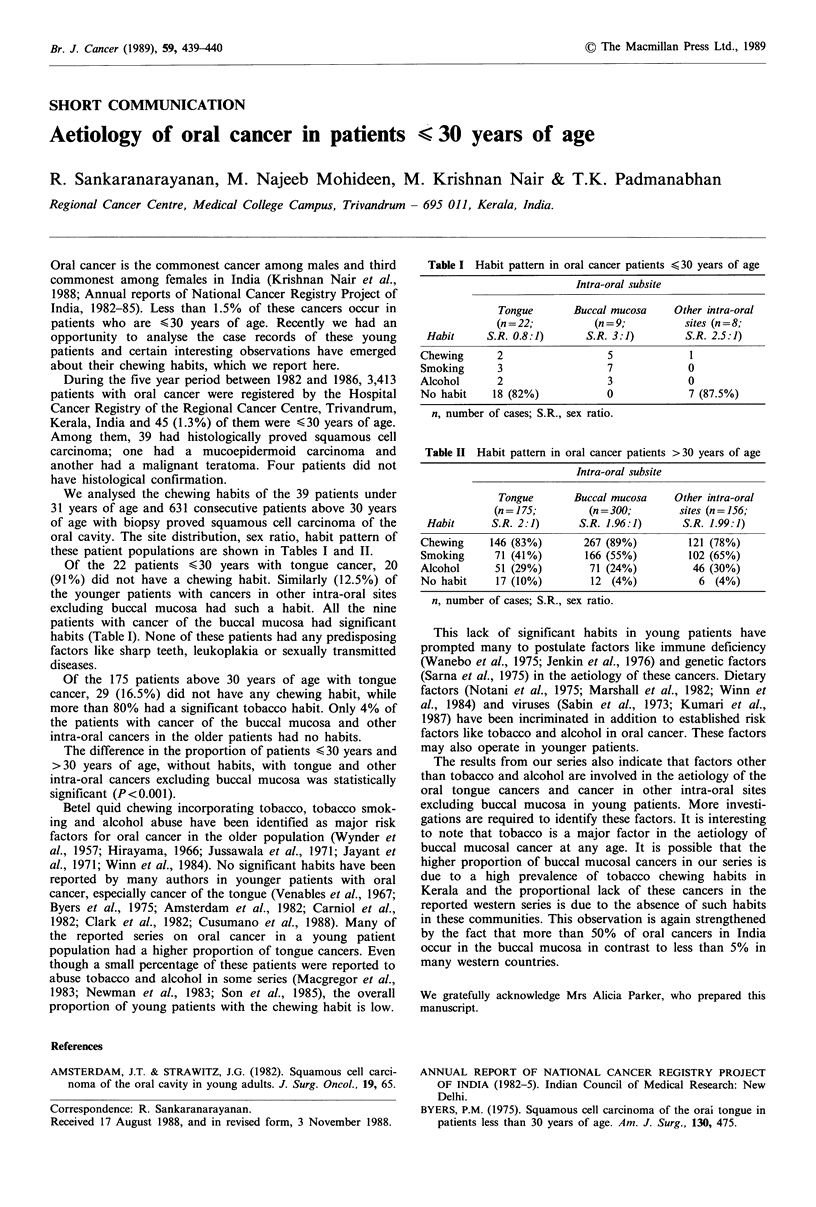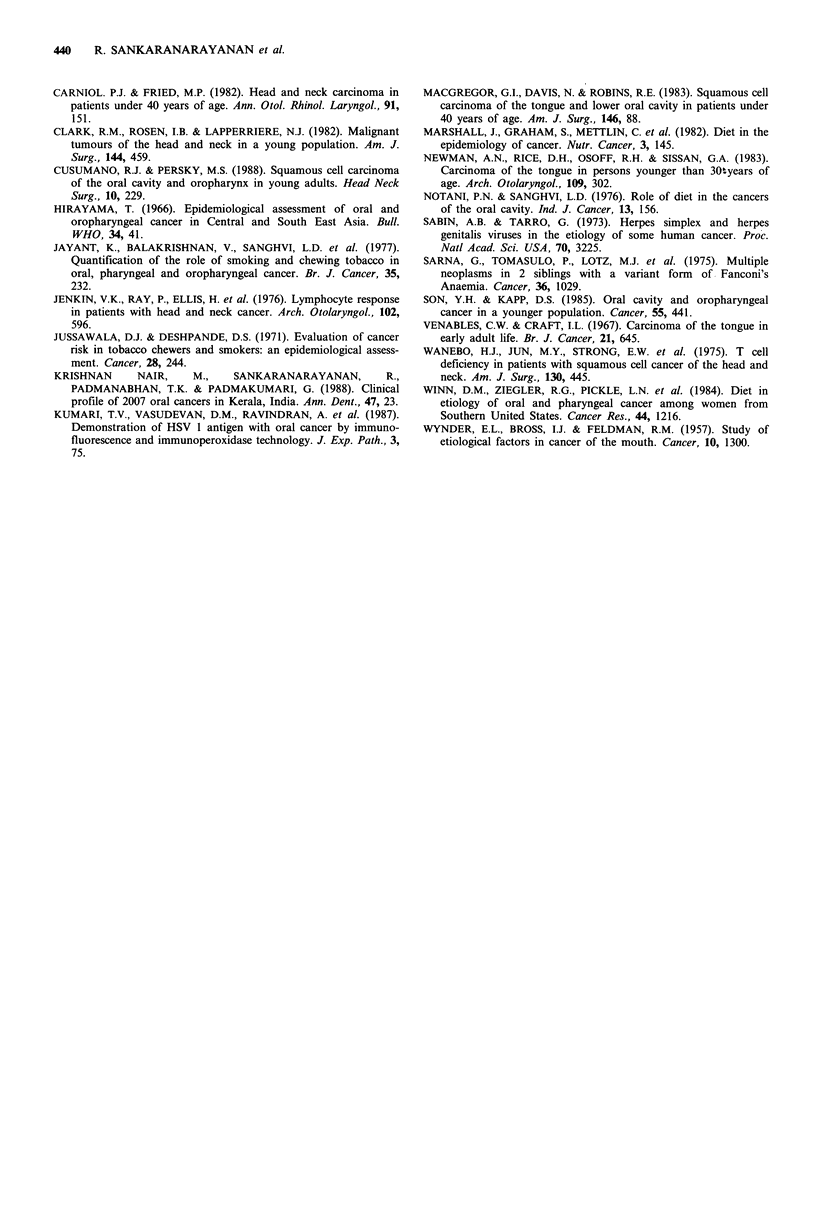# Aetiology of oral cancer in patients less than or equal to 30 years of age.

**DOI:** 10.1038/bjc.1989.89

**Published:** 1989-03

**Authors:** R. Sankaranarayanan, M. N. Mohideen, M. K. Nair, T. K. Padmanabhan

**Affiliations:** Regional Cancer Centre, Medical College Campus, Kerala, India.


Br. J. Cancer (1989), 59, 439–440 © The Macmillan Press Ltd., 1989

SHORT COMMUNICATION

### Aetiology of oral cancer in patients ≤30 years of age

R. Sankaranarayanan, M. Najeeb Mohideen, M. Krishnan Nair & T.K. Padmanabhan

*Regional Cancer Centre, Medical College Campus, Trivandrum – 695 011, Kerala, India.*

Oral cancer is the commonest cancer among males and third commonest among females in India (Krishnan Nair *et al.*, 1988; Annual reports of National Cancer Registry Project of India, 1982–85). Less than 1.5% of these cancers occur in patients who are ≤30 years of age. Recently we had an opportunity to analyse the case records of these young patients and certain interesting observations have emerged about their chewing habits, which we report here.

During the five year period between 1982 and 1986, 3,413 patients with oral cancer were registered by the Hospital Cancer Registry of the Regional Cancer Centre, Trivandrum, Kerala, India and 45 (1.3%) of them were ≤30 years of age. Among them, 39 had histologically proved squamous cell carcinoma; one had a mucoepidermoid carcinoma and another had a malignant teratoma. Four patients did not have histological confirmation.

We analysed the chewing habits of the 39 patients under 31 years of age and 631 consecutive patients above 30 years of age with biopsy proved squamous cell carcinoma of the oral cavity. The site distribution, sex ratio, habit pattern of these patient populations are shown in Tables I and II.

Of the 22 patients ≤30 years with tongue cancer, 20 (91%) did not have a chewing habit. Similarly (12.5%) of the younger patients with cancers in other intra-oral sites excluding buccal mucosa had such a habit. All the nine patients with cancer of the buccal mucosa had significant habits (Table I). None of these patients had any predisposing factors like sharp teeth, leukoplakia or sexually transmitted diseases.

Of the 175 patients above 30 years of age with tongue cancer, 29 (16.5%) did not have any chewing habit, while more than 80% had a significant tobacco habit. Only 4% of the patients with cancer of the buccal mucosa and other intra-oral cancers in the older patients had no habits.

The difference in the proportion of patients ≤30 years and >30 years of age, without habits, with tongue and other intra-oral cancers excluding buccal mucosa was statistically significant ($P<0.001$).

Betel quid chewing incorporating tobacco, tobacco smoking and alcohol abuse have been identified as major risk factors for oral cancer in the older population (Wynder *et al.*, 1957; Hirayama, 1966; Jussawala *et al.*, 1971; Jayant *et al.*, 1971; Winn *et al.*, 1984). No significant habits have been reported by many authors in younger patients with oral cancer, especially cancer of the tongue (Venables *et al.*, 1967; Byers *et al.*, 1975; Amsterdam *et al.*, 1982; Carniol *et al.*, 1982; Clark *et al.*, 1982; Cusumano *et al.*, 1988). Many of the reported series on oral cancer in a young patient population had a higher proportion of tongue cancers. Even though a small percentage of these patients were reported to abuse tobacco and alcohol in some series (Macgregor *et al.*, 1983; Newman *et al.*, 1983; Son *et al.*, 1985), the overall proportion of young patients with the chewing habit is low.

**Table I** Habit pattern in oral cancer patients ≤30 years of age

| | *Intra-oral subsite* | | |
|---|---|---|---|
| *Habit* | *Tongue (n=22; S.R. 0.8:1)* | *Buccal mucosa (n=9; S.R. 3:1)* | *Other intra-oral sites (n=8; S.R. 2.5:1)* |
| Chewing | 2 | 5 | 1 |
| Smoking | 3 | 7 | 0 |
| Alcohol | 2 | 3 | 0 |
| No habit | 18 (82%) | 0 | 7 (87.5%) |

*n*, number of cases; S.R., sex ratio.

**Table II** Habit pattern in oral cancer patients >30 years of age

| | *Intra-oral subsite* | | |
|---|---|---|---|
| *Habit* | *Tongue (n=175; S.R. 2:1)* | *Buccal mucosa (n=300; S.R. 1.96:1)* | *Other intra-oral sites (n=156; S.R. 1.99:1)* |
| Chewing | 146 (83%) | 267 (89%) | 121 (78%) |
| Smoking | 71 (41%) | 166 (55%) | 102 (65%) |
| Alcohol | 51 (29%) | 71 (24%) | 46 (30%) |
| No habit | 17 (10%) | 12 (4%) | 6 (4%) |

*n*, number of cases; S.R., sex ratio.

This lack of significant habits in young patients have prompted many to postulate factors like immune deficiency (Wanebo *et al.*, 1975; Jenkin *et al.*, 1976) and genetic factors (Sarna *et al.*, 1975) in the aetiology of these cancers. Dietary factors (Notani *et al.*, 1975; Marshall *et al.*, 1982; Winn *et al.*, 1984) and viruses (Sabin *et al.*, 1973; Kumari *et al.*, 1987) have been incriminated in addition to established risk factors like tobacco and alcohol in oral cancer. These factors may also operate in younger patients.

The results from our series also indicate that factors other than tobacco and alcohol are involved in the aetiology of the oral tongue cancers and cancer in other intra-oral sites excluding buccal mucosa in young patients. More investigations are required to identify these factors. It is interesting to note that tobacco is a major factor in the aetiology of buccal mucosal cancer at any age. It is possible that the higher proportion of buccal mucosal cancers in our series is due to a high prevalence of tobacco chewing habits in Kerala and the proportional lack of these cancers in the reported western series is due to the absence of such habits in these communities. This observation is again strengthened by the fact that more than 50% of oral cancers in India occur in the buccal mucosa in contrast to less than 5% in many western countries.

We gratefully acknowledge Mrs Alicia Parker, who prepared this manuscript.

### References

AMSTERDAM, J.T. & STRAWITZ, J.G. (1982). Squamous cell carcinoma of the oral cavity in young adults. *J. Surg. Oncol.*, **19**, 65.

Correspondence: R. Sankaranarayanan.
Received 17 August 1988, and in revised form, 3 November 1988.

ANNUAL REPORT OF NATIONAL CANCER REGISTRY PROJECT OF INDIA (1982–5). Indian Council of Medical Research: New Delhi.

BYERS, P.M. (1975). Squamous cell carcinoma of the orai tongue in patients less than 30 years of age. *Am. J. Surg.*, **130**, 475.

CARNIOL. P.J. & FRIED, M.P. (1982). Head and neck carcinoma in patients under 40 years of age. *Ann. Otol. Rhinol. Laryngol.*, **91**, 151.

CLARK, R.M., ROSEN, I.B. & LAPPERRIERE, N.J. (1982). Malignant tumours of the head and neck in a young population. *Am. J. Surg.*, **144**, 459.

CUSUMANO, R.J. & PERSKY, M.S. (1988). Squamous cell carcinoma of the oral cavity and oropharynx in young adults. *Head Neck Surg.*, **10**, 229.

HIRAYAMA, T. (1966). Epidemiological assessment of oral and oropharyngeal cancer in Central and South East Asia. *Bull. WHO*, **34**, 41.

JAYANT, K., BALAKRISHNAN, V., SANGHVI, L.D. *et al.* (1977). Quantification of the role of smoking and chewing tobacco in oral, pharyngeal and oropharyngeal cancer. *Br. J. Cancer*, **35**, 232.

JENKIN, V.K., RAY, P., ELLIS, H. *et al.* (1976). Lymphocyte response in patients with head and neck cancer. *Arch. Otolaryngol.*, **102**, 596.

JUSSAWALA, D.J. & DESHPANDE, D.S. (1971). Evaluation of cancer risk in tobacco chewers and smokers: an epidemiological assessment. *Cancer*, **28**, 244.

KRISHNAN NAIR, M., SANKARANARAYANAN, R., PADMANABHAN, T.K. & PADMAKUMARI, G. (1988). Clinical profile of 2007 oral cancers in Kerala, India. *Ann. Dent.*, **47**, 23.

KUMARI, T.V., VASUDEVAN, D.M., RAVINDRAN, A. *et al.* (1987). Demonstration of HSV 1 antigen with oral cancer by immunofluorescence and immunoperoxidase technology. *J. Exp. Path.*, **3**, 75.

MACGREGOR, G.I., DAVIS, N. & ROBINS, R.E. (1983). Squamous cell carcinoma of the tongue and lower oral cavity in patients under 40 years of age. *Am. J. Surg.*, **146**, 88.

MARSHALL, J., GRAHAM, S., METTLIN, C. *et al.* (1982). Diet in the epidemiology of cancer. *Nutr. Cancer*, **3**, 145.

NEWMAN, A.N., RICE, D.H., OSOFF, R.H. & SISSAN, G.A. (1983). Carcinoma of the tongue in persons younger than 30 years of age. *Arch. Otolaryngol.*, **109**, 302.

NOTANI, P.N. & SANGHVI, L.D. (1976). Role of diet in the cancers of the oral cavity. *Ind. J. Cancer*, **13**, 156.

SABIN, A.B. & TARRO, G. (1973). Herpes simplex and herpes genitalis viruses in the etiology of some human cancer. *Proc. Natl Acad. Sci. USA*, **70**, 3225.

SARNA, G., TOMASULO, P., LOTZ, M.J. *et al.* (1975). Multiple neoplasms in 2 siblings with a variant form of Fanconi's Anaemia. *Cancer*, **36**, 1029.

SON, Y.H. & KAPP, D.S. (1985). Oral cavity and oropharyngeal cancer in a younger population. *Cancer*, **55**, 441.

VENABLES, C.W. & CRAFT, I.L. (1967). Carcinoma of the tongue in early adult life. *Br. J. Cancer*, **21**, 645.

WANEBO, H.J., JUN, M.Y., STRONG, E.W. *et al.* (1975). T cell deficiency in patients with squamous cell cancer of the head and neck. *Am. J. Surg.*, **130**, 445.

WINN, D.M., ZIEGLER, R.G., PICKLE, L.N. *et al.* (1984). Diet in etiology of oral and pharyngeal cancer among women from Southern United States. *Cancer Res.*, **44**, 1216.

WYNDER, E.L., BROSS, I.J. & FELDMAN, R.M. (1957). Study of etiological factors in cancer of the mouth. *Cancer*, **10**, 1300.